# Advanced glycation end-products decreases expression of endothelial nitric oxide synthase through oxidative stress in human coronary artery endothelial cells

**DOI:** 10.1186/s12933-017-0531-9

**Published:** 2017-04-20

**Authors:** Xiaomei Ren, Liqun Ren, Qin Wei, Hua Shao, Long Chen, Naifeng Liu

**Affiliations:** 1 Department of Geriatrics, Zhongda Hospital, School of Medicine, Southeast University, No. 87, Dingjiaqiao Road, Nanjing, 210009 China; 2 Department of Cardiology, Zhongda Hospital, School of Medicine, Southeast University, No. 87, Dingjiaqiao Road, Nanjing, 210009 China; 3 Department of Pharmacy, Zhongda Hospital, Southeast University, No. 87, Dingjiaqiao Road, Nanjing, 210009 China; 4 School of Medicine, Southeast University, No. 87, Dingjiaqiao Road, Nanjing, 210009 China

**Keywords:** Advanced glycation end-products, Endothelial nitric oxide synthase, Oxidative stress, Endothelial dysfunction

## Abstract

**Background:**

Advanced glycation end-products (AGEs) are elevated under diabetic conditions and associated with insulin resistance, endothelial dysfunction and vascular inflammation in humans. It has been demonstrated that AGEs evoke oxidative and inflammatory reactions in endothelial cells through the interaction with a receptor for AGEs (RAGE). Here, we aimed to identify the cellular mechanisms by which AGEs exacerbate the endothelial dysfunction in human coronary artery endothelial cells (HCAECs).

**Methods:**

30 type 2 diabetic patients with or without coronary artery atherosclerosis were recruited for this study. Plasma levels of AGE peptides (AGE-p) were analyzed using flow injection assay. Endothelial function was tested by brachial artery flow-mediated vasodilatation (FMD). Further investigations were performed to determine the effects and mechanisms of AGEs on endothelial dysfunction in HCAECs.

**Results:**

AGE-p was inversely associated with FMD in diabetic patients with coronary artery atherosclerosis in our study. After treated with AGEs, HCAECs showed significant reductions of eNOS mRNA and protein levels including eNOS and phospho-eNOS Ser1177, eNOS mRNA stability, eNOS enzyme activity, and cellular nitric oxide (NO) levels, whereas superoxide anion production was significantly increased. In addition, AGEs significantly decreased mitochondrial membrane potential, ATP content and catalase and superoxyde dismutase (SOD) activities, whereas it increased NADPH oxidase activity. Treatment of the cells with antioxidants SeMet, SOD mimetic MnTBAP and mitochondrial inhibitor thenoyltrifluoroacetone (TTFA) effectively blocked these effects induced by AGEs. AGEs also increased phosphorylation of the mitogen-activated protein kinases p38 and ERK1/2, whereas the specific inhibitors of p38, ERK1/2, and TTFA effectively blocked AGEs-induced reactive oxygen species production and eNOS downregulation.

**Conclusions:**

AGEs cause endothelial dysfunction by a mechanism associated with decreased eNOS expression and increased oxidative stress in HCAECs through activation of p38 and ERK1/2.

**Electronic supplementary material:**

The online version of this article (doi:10.1186/s12933-017-0531-9) contains supplementary material, which is available to authorized users.

## Background

Coronary artery atherosclerosis is very common in diabetes mellitus and its presence predicts future cardiovascular events. Endothelial dysfunction is a key early event in atherogenesis [1, 2]. Normal endothelial cells constitutively express endothelial nitric oxide synthase (eNOS), which plays a critical role in maintaining the endothelial functions including regulation of vascular tone, barrier function, inhibition of coagulation and thrombosis, suppression of inflammatory cell adhesion and migration, and angiogenesis. Endothelium-derived NO, synthesized by eNOS, is a major mediator of endothelium-dependent vasorelaxation [3]. Endothelial dysfunction has largely been assessed as alterations of endothelium-dependent vasorelaxation and gene expression. Many cardiovascular risk factors induce endothelial dysfunction through impairment of eNOS–NO system, which likely explains their promotion of atherogenesis.

Recently, researches suggest that AGEs, senescent macroprotein derivatives formed at an accelerated rate in diabetes, promotes the development of endothelial dysfunction in diabetic patient [4, 5]. AGEs can bind to its receptor RAGE, triggering the production of several proinflammatory cytokines and chemokines, inducing oxidative stress via the activation of NF-kB [6]. AGEs was able to reduce NO production and eNOS expression under the condition of high glucose [5]. AGEs also increases ROS production by activation of NADPH oxidase [7, 8]. AGE inhibitor, aminoguanidine, can improve endothelial function in diabetic animal models [1, 4]. Despite accumulating evidence pointing to a causal role of AGEs-induced lesion in the pathogenesis of diabetic vascular disorder, the molecular mechanisms involved in endothelial dysfunction remains poorly understood.

Here we took advantage of HCAECs as a model to investigate the effects of AGEs on eNOS-NO system as well as its related mechanisms including oxidative stress, and MAPK activation involvement. The relationship between serum levels of AGE-p and flow-mediated vasodilatation (FMD) in diabetic patients with coronary artery atherosclerosis were also determined. This study may provide new insights into the mechanisms of AGEs interacting with endothelial cells that may contribute to the vascular lesion formation.

## Methods

### Cell culture

HCAECs and endothelial growth medium-2 were purchased from Cambrex BioWhittaker (Walkersville, MD). Cells were cultured at 37 °C in 5% carbon dioxide (CO_2_) and detached by incubation with 0.25% trypsin–EDTA solution. Passages 5–6 were used in this study. AGEs were prepared as described previously [9]. Briefly, 5 g of bovine serum albumin (BSA) was incubated with 9 g of d-glucose in 100 ml sodium phosphate buffer (PBS) at 37 °C for 90 days, and finally was dialyzed against PBS. As a control, BSA was incubated in parallel without d-glucose. No endotoxin was detectable in these preparations.

### Subjects

15 type 2 diabetic patients (average age 63.6 years range 41–74 years; 11 men, 4 women) with coronary artery atherosclerosis scheduled for percutaneous coronary intervention at Zhongda hospital affiliated with the Southeast University were recruited for this study. 15 type 2 diabetic patients (average age 58.9 years range 43–77 years; 10 men, 5 women) without coronary artery atherosclerosis were enrolled as control. All patients received insulin injection to control blood glucose. Serum AGE-p was analyzed using flow injection assay. The protocol was approved by the Ethic Committee of the Zhongda Hospital affiliated with the Southeast University and the methods used in this study were carried out in accordance with the approved guidelines. The informed consent was obtained from all patients.

### Measurements of endothelial function

Brachial artery FMD was used to test endothelial function. Methodology and reproducibility data have been described previously [10]. Briefly, the brachial artery above the elbow was scanned in the supine position by use of high-resolution ultrasound at rest and 1 min after reactive hyperemia that was induced by 5-min cuff occlusion of forearm blood flow. The baseline diameter and maximum FMD diameter were measured from one media-adventitia interface to the other by commercially-available edge-detection software. Vasodilatarion was then calculated as the percent change in diameter over the baseline value.

### Gene expression analysis

Total RNA was isolated from HCAECs with TRIzol reagent (Invitrogen Carlsbad, CA) according to the manufacturer’s instructions. 50 ng of RNA were converted to cDNA using the iScript cDNA Synthesis Kit (Bio-Rad, Hercules, CA). Glyceraldehyde-3-phosphatede hydrogenase was used as housekeeping gene to account for variations in mRNA loading. PCR amplification was performed using SYBR Green PCR master mix (Applied Biosystems, Foster City, CA) according to the manufacturer’s instructions. To assess the mRNA stability or half-life of eNOS mRNA, HCAECs were treated with 5 μg/ml actionmycin D with or without AGEs (100 mmol/l).

### Antibodies and immunoblotting

Equal amounts of endothelial proteins (6 g) were resolved using SDS–polyacrylamide gel electrophoresis, and then transferred onto nitrocellulose membranes according to standard procedures. Immunoblot analysis was carried out using antibodies directed against β-actin (Sigma), eNOS, phospho-eNOS Ser1177 (BD Biosciences, SanJose, CA), phospho- and total extracellular signal-regulated kinase (ERK)1/2, c-Jun NH2-terminal kinase (JNK), and p38 (RD systems, Minneapolis, MN).

### eNOS enzyme activity

A fuorometric cell-associated NOS detection system (Sigma) was used to measure intracellular production of nitric oxide (NO) from supplemented l-arginine by a nonradiometric method.

### Nitrite detection

NO levels in HCAECs supernatants were determined by measuring the levels of nitrite and nitrate, the stable degradation products of NO (Griess reaction NO assay kit; Calbiochem). Total amount of nitrite in HCAECs was determined and normalized to total proteins of HCAECs (pmol/mg protein).

### Cellular NO levels and reactive oxygen species (ROS) production assay

HCAECs were harvested and adjusted to (1 × 10^6^/ml) cells per each FACS tube. For cellular NO staining, treated cells were incubated with 4-amino-5-methylamino-2′, 7′-difluorofluorescein diacetate (DAF, 10 μM; Molecular Probes) at 37 °C for 30 min and then washed. Flow cytometry assay was used to measure the stained cells. ROS levels were studied with dihydroethidium (DHE, 5 μM; Molecular Probes) staining and flow cytometric analysis. Samples were analyzed using FACScan and Cell Quest software (Becton–Dickinson, Franklin Lakes, NJ). Mitochondrial membrane potential was determined with 5,5′,6,6′-tetrachloro-1,1′,3,3′-tetraethylbenzimidazole-carbocyanide iodine (JC-1, MitoScreen kit; BD Biosciences) staining and flow cytometric analysis. ATP levels in HCAECs were measured with an ATPLite kit (Perkin–Elmer, Waltham, MA) per manufacturer’s instructions. HCAECs were cultured and treated as previously described on 6 well plates for 24 h. The lysis and substrate solutions were added to each well. The luminescence was measured by the TopCount Microplate Scintillation and Luminescence Counter (Perkin-Elmer, Waltham, MA).

### Cellular glutathione assay

GSH-Glo Glutathione assay (Promega, Madison, WI) measures a change in the redox state of the cell due to oxidants, which are downstream metabolites of O_2_^−^.

### Measurement of NAPDH oxidase, CAT and SOD activities

NAPDH oxidase was determined by lucigenin-enhanced chemiluminescence method (Promega, Madison, WI). CAT and SOD enzyme activities were measured with commercial enzyme assay kits (Cay-man Chemical, Ann Arbor, MI) according to the manufacturer’s protocols.

### BioPlex immunoassay

HCAECs were cultured with 100 μg/ml of AGEs for 0, 5, 10, 20, 30, 45, 60, or 90 min. Cell lysate was prepared. Detection of phospho- and total extracellular signal-regulated kinase (ERK) 1/2, c-Jun NH2-terminal kinase (JNK), and p38 was performed by the BioPlex Luminex system 2200 (Bio-Rad).

### Statistical analysis

Data are reported as mean ± SD of at least triplicate determinations. Statistical significance (*P* < 0.05) was determined by paired Student’s t test (Statview, Abacus Concepts, Berkeley, CA).

## Results

### AGE-p was inversely associated with FMD in type 2 diabetic patients with coronary artery atherosclerosis

**Fig. 1:**
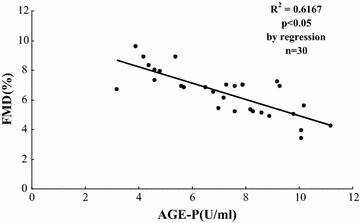
Correlations between plasma level of AGE-p and FMD in type 2 diabetic patients with or without coronary artery disease. AGE-p: advanced glycation end product peptides (U/ml); FMD: flow-mediated vasodilatation (%)

To investigate of relationship between AGE-p and FMD in type 2 diabetic patients with or without coronary artery atherosclerosis, levels of plasma AGE-p and flow-mediated vasodilatation were tested. As shown in Table 1, plasma levels of AGE-p were at high level; FMD was at low level. In contrast, plasma levels of AGE-p were at low level; FMD was at high level. These result showed that the levels of plasma AGE-p were inversely associated with flow-mediated vasodilatation in our subjects (R^2^ = −0.61, P < 0.05), whereas other cardiometabolic risk factors, including age, fasting plasma glucose, HbA_1_C, blood pressure, lipid parameters and body mass index, were not associated with FMD. In addition, AGE-p was at high levels in diabetic patients with coronary artery atherosclerosis, compared with diabetic patients without coronary artery atherosclerosis (P < 0.05, Fig. 1). These result showed that AGE-p was the risk factor of coronary artery atherosclerosis.

**Table 1:** Clinical characteristics of the subjects

	Diabetes with CAD	Diabetes without CAD
Age (years)	59.2 ± 7.9	58.3 ± 9.9
Sex (number, male/female)	11/4	10/5
Body mass index (kg/m^2^)	24.3 ± 1.2	23.1 ± 1.5
AGE-p (U/ml)	6.3 ± 1.5	5.1 ± 0.9*
Systolic blood pressure (mmHg)	130.2 ± 16.2	133.2 ± 22.1
Diastolic blood pressure (mmHg)	81.2 ± 13.2	85.2 ± 10.2
Total cholesterol (mmol/l)	4.22 ± 0.54	4.56 ± 0.26
HDL cholesterol (mmol/l)	1.08 ± 0.27	0.98 ± 0.19
Triglycerides (mmol/l)	1.96 ± 0.41	2.01 ± 0.19
Fasting glucose(mmol/l)	6.48 ± 0.7	6.2 ± 0.3
HbA 1c (%)	6.4 ± 0.76	6.7 ± 0.26
Serum creatinine (μmol/l)	68.2 ± 17.9	71.2 ± 14.8
Microalbuminuria/urine creatinin	42.3 ± 4.5	35.6 ± 9.2

^*^P < 0.05 compared with diabetes with CAD

*AGE-p* advanced glycation end products-peptides, *HDL* high-density lipoprotein, *CAD* coronary artery atherosclerosis

### AGEs decreases the levels of eNOS and NO expression in HCAECs

**Fig. 2:**
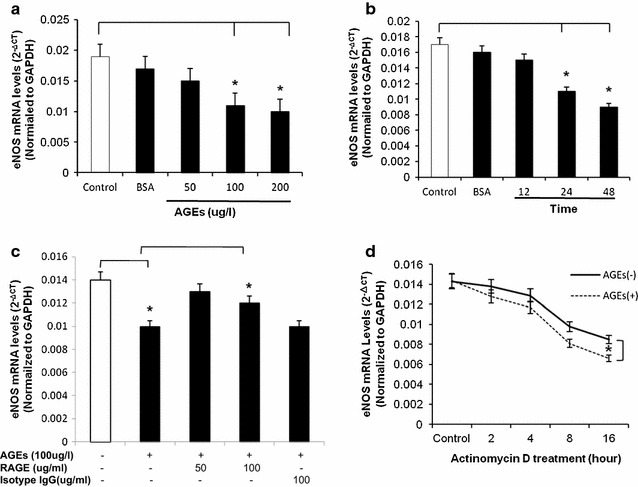
Effects of AGEs on eNOS mRNA in HCAECs. HCAECs were cultured with different concentrations of AGEs for different periods of time. The mRNA levels of eNOS and glyceraldehyde-3-phosphatede-hydrogenase (GAPDH) were determined by real-time PCR analysis. **a** Concentration-dependent study. Cells were treated with different concentrations of AGEs (50, 100, or 200 μg/ml) for 24 h. **b** Time-dependent study. Cells were treated with AGEs (100 μg/ml) for different times (12, 24 and 48 h). **c** Effect of anti-RAGE antiboday. Cells were treated with 100 μg/ml AGEs and different concentrations of anti-RAGE antiboday for 30 min and followed with AGEs treatment for 24 h. Isotype IgG was used for a negative control. **d** eNOS mRNA stability. Cells were treated with 5 μg/ml actinomycin D in the presence or absence of AGEs (100 μg/ml) for indicated time points (0, 2, 4, 8, or 16 h), and eNOS mRNA levels were determined by real-time PCR. * *P* < 0.05, compare with control, ^**#**^ *P* < 0.05, compare with AGEs treatment, n = 3 experiments. Data are means and SE of multiple experiments (n)

The expression of eNOS and NO was evaluated after HCAECs were treated with AGEs in a concentration- and time-dependent manner. eNOS mRNA and protein levels were detected using real-time PCR and Western blot, respectively. When cells were treated with AGEs (100 or 200 μg/ml) for 24 h, eNOS mRNA levels were decreased by 31 and 41%, respectively, compared with controls (*P* < 0.05, Fig. 2a). Treatment with BSA (100 μg/ml) alone did not cause any decrease in eNOS mRNA levels, compared with controls in HCAECs (*P* < 0.05, Fig. 2a).

For time-dependent experiment, cells were cultured with AGEs (100 μg/ml) for 12, 24 and 48 h. The results showed that when cells were treated with AGEs for 24 and 48 h, eNOS mRNA levels were decreased by 33 and 45%, respectively, compared with controls (*P* < 0.05, Fig. 2b). To further determine the specific effect of AGEs on eNOS expression, HCAECs were treated with AGEs (100 μg/ml) and anti-RAGE antibody (RAGE, receptor of AGEs) (50 or 100 μg/ml), or isotype IgG (100 μg/ml) antibody for 24 h. 100 μg/ml RAGE significantly blocked the decrease in eNOS induced by AGEs (*P* < 0.05, Fig. 2c). Isotype antibody as negative control at the same concentration showed no effect on the AGEs-induced eNOS mRNA decrease (Fig. 2c). By using actinomycin D, a direct inhibitor of RNA polymerase II, 100 ng/ml AGEs also showed the decrease in eNOS mRNA stability in HCAECs, compared with control (*P* < 0.05, Fig. 2d). The half-life of eNOS mRNA decreased from >16 h in control cells to <8 h in AGEs-treated HCAECs.

**Fig. 3:**
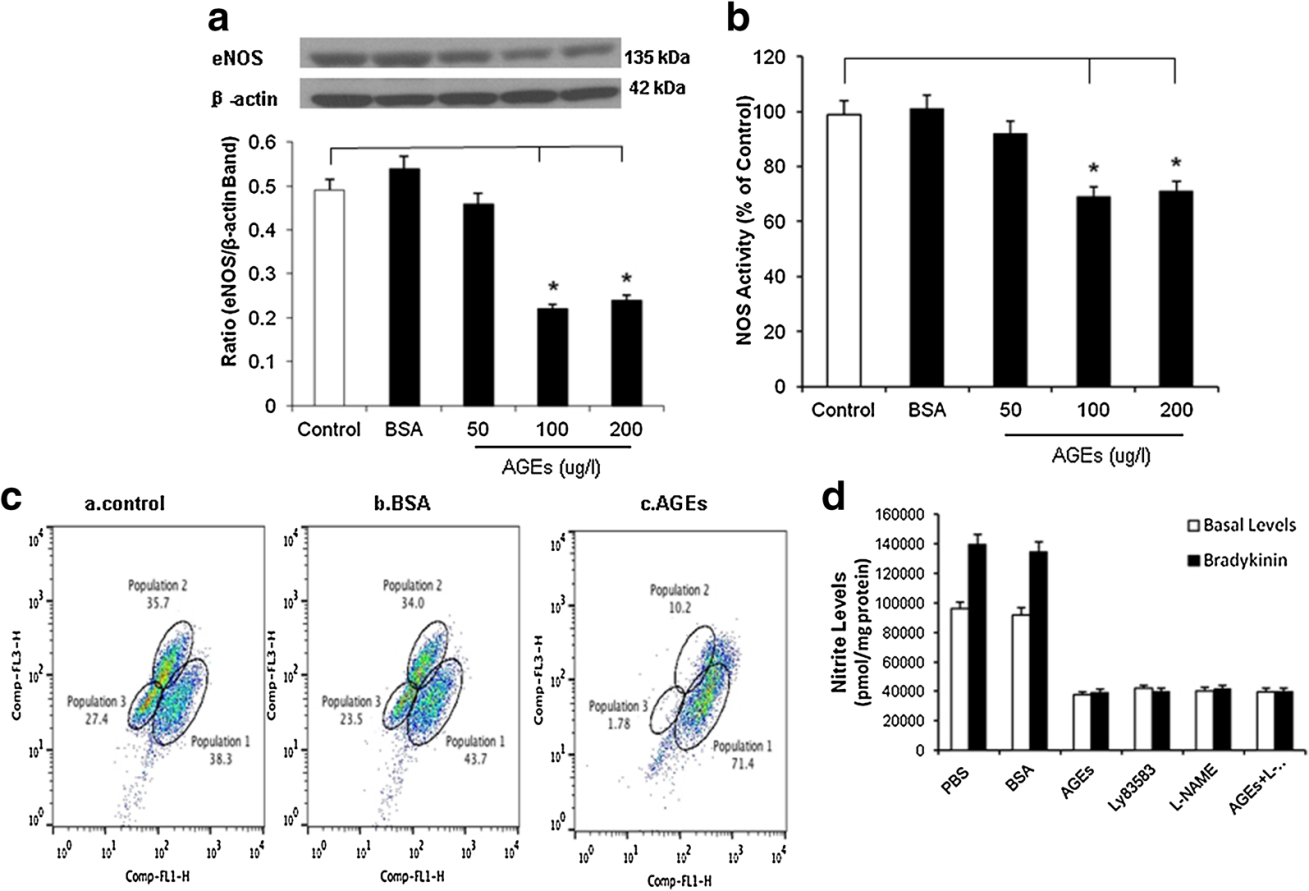
Effects of AGEs on eNOS protein levels and NOS activity in HCAECs. **a** Western blot analysis. Cells were treated with 50, 100 or 200  μg/ml AGEs for 24 h. Representative bands of eNOS and β-actin staining and quantitation of band density ratios (eNOS and β-actin) Full-length blots are presented. *P < 0.05 compare with control, *n* = 3 experiments. Data are means and SE of multiple experiments (n). **b** NOS activity. Cells were treated withs AGEs for 24 h. The NOS activity was determined by a commercial eNOS fluorimetric assay kit. **c** Cellular NO levels. Cells were treated with AGEs (100 μg/l) for 24 h and cellular NO levels were determined by DAF-FM DA (10 μM) staining and flow cytometric analysis. **d** Nitric oxide (NO) levels in the supernatant of cell culture (Griess assay). Cells were treated with AGEs (100 μg/ml) in the presence or absence of LY-83,583 (3 μM) or l-NAME (100 μM) for 24 h. Basal and bradykinin-stimulated levels of NO-derived nitrite in the culture supernatant were detected. * *P* < 0.05, compare with control, n = 3 experiments. Data are means and SE of multiple experiments (n)

Western blot showed that HCAECs were treated with AGEs at 100 and 200 μg/ml, eNOS protein levels were significantly decreased by 29 and 41%, respectively, compared with controls (*P* < 0.05, Fig. 3a). P-eNOS Ser1177 phosphorylation in HCAECs treated with AGEs at 100 μg/ml for 24 h was also decreased by 32% compared with control (*P* < 0.05, Additional file 1: Fig. S1).

NOS activities were studied using a NOS detection system. In line with the results of real-time PCR and Western blot, after treated with 100 and 200 μg/ml AGEs, NOS activity was significantly decreased by 25 and 36% respectively, compared with controls in HCAECs (*P* < 0.05, Fig. 3b).

Cellular NO levels in HCAECs were also measured using the fluorescent dye DAF. DAF staining is a unique method measuring NO production in living cells or solutions [9]. HCAECs were treated with different concentration of AGEs for 24 h, and then incubated with 10 μM of DAF for 30 min. Flow cytometry assay was used to measure the stained cells. AGEs at 100 and 200 μg/ml decreased the number of NO positive cell by 18 and 31%, respectively, compared with controls (*P* < 0.05, Fig. 3c). Furthermore, nitrite levels derived by NO in the supernatants of HCAECs were studied by using of Griess assay. LY-83583, a known superoxide generator, was taken as a positive control in our experiments. The results showed that both basal and bradykinin stimulated levels of NO in HCAECs were significantly decreased after AGEs treatment at 100 μg/ml for 24 h. The combination of specific eNOS inhibitor l-NAME (100 μM) with AGEs did not cause further reduction in NO levels compared with AGEs treatment alone in HCAECs. Meanwhile, in HCAECs treated with LY-83583 (3 μM) for 24 h, NO levels showed a decrease, which was similar to that seen in AGEs-treated cells (*P* < 0.05, Fig. 3d). These data indicate that AGEs specifically inhibits eNOS.

### AGEs increases ROS production in HCAECs

**Fig. 4:**
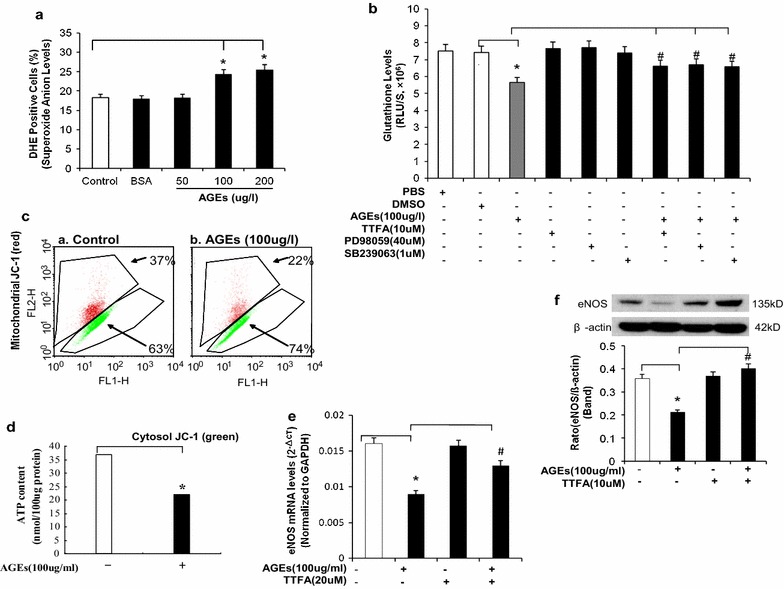
Effects of AGEs on oxidative stress in HCAECs. **a** O_2_^−^ levels. Cells were treated with 50, 100 or 200 μg/ml of AGEs for 24 h, and intracellular O_2_^−^ levels were determined by DHE (5 μM) staining and flow cytometric analysis. **b** ROS production (glutathione assay). HCAECs were treated with AGEs (100 μg/ml) and/or other molecules for 24 h. RLU, relative light units; TFA, thenoyltrifluoroacetone. **c** Mitochondrial membrane potential (JC-1 staining and flow cytometry). Cells were treated with AGEs for 24 h, and mitochondrial membrane potential was determined by JC-1 staining and flow cytometric analysis. **d** ATP content. Cells were treated with 100 μg/ml AGEs for 24 h, and ATP content was determined by an ATPLite kit. * *P* < 0.05, compare with control. **e** Effect of mitochondrial complexII inhibitor TTFA on eNOS mRNA levels. HCAECs were treated with AGEs and/or TFA (10 μM) for 24 h, and eNOS mRNA levels were determined by real-time PCR analysis. F: Effect of mitochondrial complexII inhibitor TTFA on eNOS protein levels. HCAECs were treated with AGEs and/or TTFA (10 μM) for 24 h, and eNOS protein levels were determined by Western blot. Full-length blots are presented. * *P* < 0.05, compare with control, ^**#**^ *P* < 0.05, compare with AGEs treatment, n = 3. Data are means and SE of multiple experiments (n)

ROS production in HCAECs was measured using fluorescence dye DHE staining and flow cytometry analysis. After treatment with AGEs (100 and 200 μg/ml) for 24 h, ROS production in HCAECs was increased substantially by 39 and 56%, respectively, compared with controls (*P* < 0.05, Fig. 4a). However, BSA (100 μg/ml) did not lead to any increase in DHE staining. Furthermore, we measured the effect of AGEs on redox state in HCAECs using GSH assay. AGEs treatment at 100 μg/ml for 24 h led to significantly decreased GSH levels compared with controls (*P* < 0.05, Fig. 4b), indicating oxidative stress.

### AGEs decreases mitochondrial membrane potential in HCAECs

The membrane potential was known to serve as an indicator of mitochondrial respiratory chain function. Because mitochondria are not only a major source of ROS, but also are particularly susceptible to oxidative damage caused by the action of ROS on lipids, proteins, and DNA. We next detected the mitochondrial membrane potential in HCAECs using JC-1 staining and flow cytometry analysis. Cells were seeded on six-well plates and cultured with or without AGEs (100 μg/ml) for 24 h. Treatment with AGEs significantly reduced mitochondrial membrane potential by 48% compared with controls (*P* < 0.05, Fig. 4c). Furthermore, 100 μg/ml AGEs decreased ATP levels in HCAECs by 29% (*P* < 0.05, Fig. 4d).

### AGEs increases NOX activity, whereas it decreases CAT and SOD activities in HCAECs

**Fig. 5:**
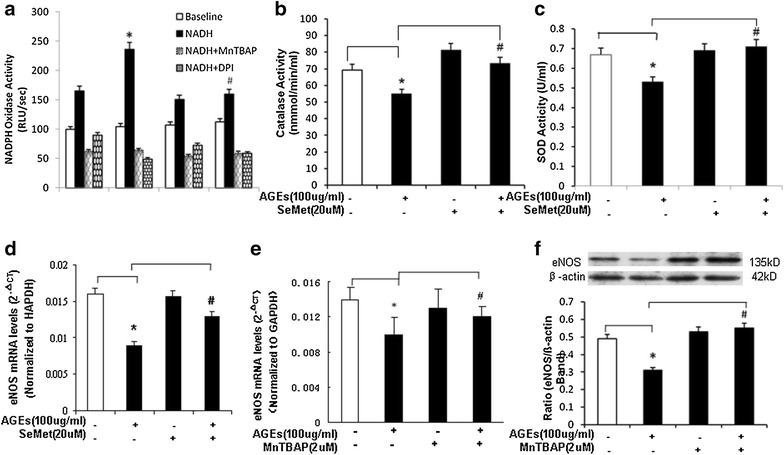
Effects of AGEs, SeMet and MnTBAP on activities of NOX, CAT, and SOD and eNOS mRNA levels in HCAECs. **a** Effect of SeMet on NADPH oxidase (NOX) activity. Cells were treated with AGEs (100 μg/ml) for 24 h, and NOX activities were determined by lucigenin-enhanced chemiluminescence with the presence of its substrate-NADPH. O_2_^−^ scavenger Tiron or flavoprotein inhibitor DPI was included in the assay to confirm the specificity of NOX activity. **b** Effect of SeMet on CAT activity. Cells were treated with AGEs (100 μg/ml) and/or antioxidant SeMet (20 μM) for 24 h and CAT activity was determined with acommercial kit. **c** Effect of SeMet on SOD activity. Cells were treated with AGEs (100 μg/ml) and/or antioxidant SeMet (20 μM) for 24 h and SOD activity was determined with acommercial kit. **d** Effect of SeMet on eNOS mRNA levels. Cells were treated with AGEs (100 μg/ml) and/or antioxidant SeMet (20 μM) for 24 h, and eNOS mRNA levels were determined by real-time PCR analysis. **e** Effect of MnTBAP on eNOS mRNA levels. Cells were treated with AGEs (100 μg/ml) and/or MnTBAP (2 μM) for 24 h, and eNOS mRNA levels were determined by real-time PCR analysis. **f** Effect of MnTBAP on eNOS protein levels. Cells were treated with AGEs (100 μg/ml) and/or MnTBAP (2 μM) for 24 h, and eNOS protein were determined by western blot. Full-length blots are presented. * *P* < 0.05, compare with control, ^**#**^ *P* < 0.05, compare with AGEs treatment, n = 3. Data are means and SE of multiple experiments (n)

In addition to mitochondrial dysfunction, ROSs can be generated from other enzymatic sources such as increased levels and/or activities of NADPH oxidase (NOX) as well as decreased levels and/or activities of internal antioxidant enzymes including superoxide dismutase (SOD) and catalase (CAT). In the current study, we determined NOX activity by chemiluminescence assay. HCAECs were treated with AGEs (100 μg/ml) and/or SeMet (20 μM) for 24 h. With the presence of β-NADPH, the NOX activity in AGEs-treated cells showed a 37% increase compared with controls (P < 0.05, Fig. 5a). O_2_^−^ scavenger MnTBAP or fiavoprotein inhibitor DPI coculture abolished this AGEs-induced increase of NOX activity, suggesting the specificity of the assay system for NOX activity. Antioxidant SeMet coculture also significantly blocked this AGEs-induced change to the control levels (P < 0.05, Fig. 5a).

The enzyme activities of CAT and SOD were studied with commercial assay kits. Treatment with AGEs (100 μg/ml) significantly reduced CAT activities by 23%, compared with controls (P < 0.05, Fig. 5b). Similarly, SOD activities were also reduced by 30% (P < 0.05, Fig. 5c). This indicates that mitochondria contribute to AGEs-induced ROS production in HCAECs.

### Antioxidants SeMet, SOD mimetic MnTBAP and mitochondrial inhibitor thenoyltrifluoroacetone (TTFA) block eNOS downregulation induced by AGEs in HCAECs

To further confirmed oxidative stress is involved in the reduction in eNOS mRNA induced by AGEs in HCAECs, antioxidant SeMet, SOD mimetic MnTBAP and TTFA were used in the next study. HCAECs were cocultured with AGEs (100 μg/ml) and antioxidant SeMet (20 μM) for 24 h. Comparing to AGEs treated alone, the addition of SeMet significantly blocked the eNOS mRNA decrease caused by AGE in HCAECs (P < 0.05, Fig. 5d). Although SeMet alone showed no effect on eNOS expression, AGEs-induced decreases of cellular CAT and SOD activities were significantly blocked by SeMet (P < 0.05, Fig. 5b, d).

Next we determine the effect of the specific O_2_^−^ scavenger MnTBAP on eNOS downregulation induced by AGEs in HCAECs, cells were treated with AGEs (100 μg/ml), MnTBAP (2 μM), or a combination of both for 24 h. Then real-time PCR and Western blot analysis were used to determine the levels of both eNOS mRNA and protein, respectively. MnTBAP effectively blocked AGEs-induced eNOS mRNA downregulation at both mRNA and protein levels (*P* < 0.05, Fig. 5e, f).

To confirm the mitochondrial source of AGEs-induced ROS, TTFA was used in the next study. HCAECs were treated with AGEs (100 μg/ml), TTFA (10 μM), or a combination of both for 24 h, ROS production was detected by GSH assay. AGEs reduced GSH levels, whereas TTFA effectively blocked the effect of AGEs in HCAECs (*P* < 0.05, Fig. 4b). In addition, TTFA (10 μM) also effectively blocked the downregulation of both eNOS mRNA and protein (*P* < 0.05, Fig. 4e, f).

### AGEs induces MAPK p38 and ERK1/2 activation in HCAECs

**Fig. 6:**
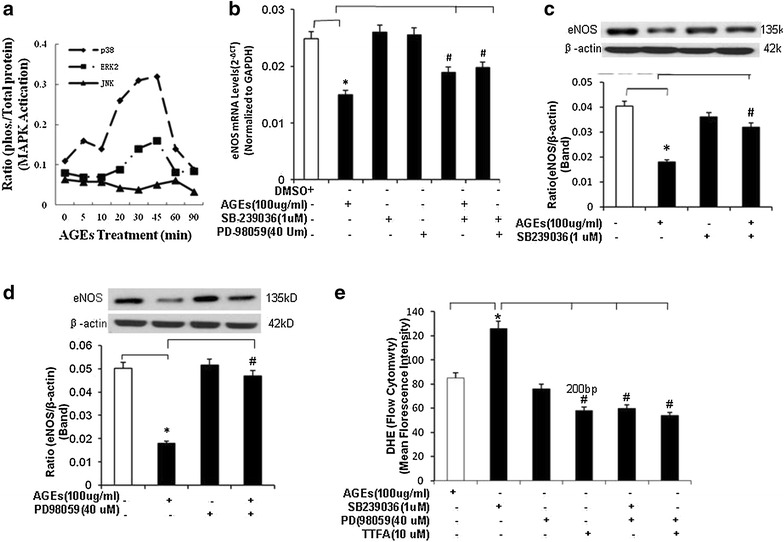
Effects of AGEs on activation of MAPKs in HCAECs. **a** MAPK p38 and ERK1/2 phosphorylation. Cells were treated with AGEs (100 μg/ml) for 24 h, then phosphorylation of MAPKs [p38, c-Jun NH2-terminal kinase (JNK), and extracellular signal-regulated kinase (ERK) 2] by using Bio-Rad Bioplex luminex immunoassay. HCAECs were treated with AGEs (100 ug/ml) for different time points. **b** Effects of MAPK inhibitors on eNOS mRNA levels. Cells were treated with AGEs (100 μg/ml) in the presence or absence of p38 inhibitor (SB239036, 1 μM) or ERK1/2 inhibitor (PD98059, 40 μM) for 24 h, and eNOS mRNA levels were determined by real-time PCR analysis. **c** Effect of p38 inhibitor (SB239036) on eNOS protein levels. Cells were treated with AGEs (100 μg/ml) in the presence or absence of p38 inhibitor (SB239036, 1 μM) for 24 h, and eNOS protein levels were determined by Western blot. Full-length blots are presented. **d** Effect of ERK1/2 inhibitor (PD98059) on eNOS protein levels. Cells were treated with AGEs (100 μg/ml) in the presence or absence of ERK1/2 inhibitor (PD98059, 40 μM) for 24 h, and eNOS protein levels were determined by Western blot. Full-length blots are presented. **e** Effects of AGEs (100 μg/ml), MAPK inhibitors, and TTFA on O_2_^−^ production. Cells were treated with AGEs (100 μg/ml) in the presence or absence of MAPK inhibitors (p38 and ERK2) or TTFA for 24 h. O_2_^−^ production was determined by DHE staining and flow cytometric analysis. *P < 0.05 compared with the control. ^#^P < 0.05 compared with AGEs treatment. n = 3. Data are means and SE of multiple experiments (n)

It is known that ERK1/2 and p38 pathways are involved in AGEs-RAGE dependent signaling [10]. In order to identify whether MAP-kinase pathways is involved. Phosphorylation of MAPKs was investigated with a BioPlex immunoassay. After AGEs treatment (100 μg/ml), there were an early phosphorylated p38 peak at 5–10 min and a second peak at 30–45 min. ERK1/2 activation only showed a single peak at 45 min after the treatment (Fig. 6a). However, JNK did not show any significant changes at any time points (Fig. 6a).

To further confirm MAP-kinase pathways involved, we performed blocking experiments with specific inhibitors of the ERK1/2 and p38 pathways. p38 inhibitor (SB-239036) or ERK inhibitor (PD-98059) were used to confirm the functional role of p38 and ERK1/2 activation in the action of AGEs. HCAECs were pretreated with SB-239036 (1 μM) or PD-98059 (40 μM) for 1 h, then cultured with AGEs (100 μg/ml) for 24 h. The eNOS mRNA and protein levels were detected using real-time PCR and western blot, respectively. Pretreatment with SB-239036 effectively blocked the AGEs-induced eNOS mRNA and protein decrease, respectively (*P* < 0.05, *n* = 3, Fig. 6b, c). In line with the effects of SB-239036 on eNOS mRNA and protein induced by AGEs, PD-98059 also effectively blocked the AGEs-induced eNOS mRNA and protein decrease, respectively (*P* < 0.05, Fig. 6b,c).

In addition, effects of MAPK inhibitors and TTFA on AGEs-induced oxidative stress were also determined with DHE staining and flow cytometry analysis. SB239036, PD98059, and TTFA effectively blocked AGEs induced increase O_2_^−^ production (*P* < 0.05, Fig. 6e). Thus, our data indicate AGEs causes a decrease in eNOS activity that may be mediated by p38 and ERK1/2.

## Discussion

In the present study, we found that plasma AGEs level was inversely correlated with endothelial function in type 2 diabetic patients with coronary artery atherosclerosis. The findings have extended the previous observations showing that serum AGEs level was correlated with endothelial dysfunction in diabetes. Moreover, we also present evidence that AGEs are able to induce endothelial dysfunction in HCAECs. Specifically, AGEs significantly reduce eNOS expression level and NOS activity as well as NO bioavailability in HCAECs. In addition, AGEs directly induce oxidative stress and activation of p38 and ERK MAPKs in HCAECs. This study provides insight into the biological functions and molecular mechanisms of AGEs in the vascular system. Consistent with previous reports [11], we observed increased serum level of AGEs in diabetes mellitus with coronary artery atherosclerotic stenosis.

Our previous study demonstrates that AGEs can increase the expression of its receptor RAGE on human umbilical vein endothelial cells (HUVECs) and rat vascular smooth muscle cells (VSMCs) through which AGEs could enhance its biologic functions in the vascular system. AGEs showed a decrease of eNOS mRNA, protein levels in HUVECs, and the eNOS enzyme activity was also decreased.

eNOS plays a critical role in maintaining the endothelial functions. Normal endothelial cells constitutively express eNOS, which catalyzes the production of NO from l-arginine. NO directly mediates vasorelaxation and many other biological processes [12]. However, many cardiovascular risk factors induce eNOS dysfunctional or decrease eNOS expression [13]. These changes of eNOS not only impair endothelium-dependent vasorelaxation but also accelerate the atherosclerotic lesion formation [14]. Growing evidence from preclinical and clinical studies has implicated that accumulation of advanced glycation end products, reactive oxygen species overproduction, and endoplasmic reticulum stress may cause the vascular structure changes through vascular endothelial growth factor A/phosphoinositide 3′ kinase/AKT/endothelial nitric oxide synthase and in the activation of antiangiogenic signals [15]. Matsui et al. reported that AGEs could elicit ROS generation and inflammatory and thrombogenic reactions in HUVECs [16]. Jo-Watanabe et al. demonstrates that age-related endothelial glycative altered phosphorylation of eNOS, and attenuated endothelial dysfunction through modulation of endothelial nitric oxide synthase phosphorylation [17].

In the present study, we demonstrated a direct effect of AGEs on cultured HCAECs. AGEs at high plasma concentrations in diabetic individuals could repress eNOS expression and activity in a time- and concentration-dependent manner, which also confirmed by anti-RAGE antibody blocking experiment. LY-83583 (O_2_^−^ generating molecule) was also used as a positive control in this study to confirm the role of O_2_^−^ in eNOS expression. Indeed, treatment with LY-83583 led to a decrease in NO levels in HCAECs, which was similar to that seen in AGEs-treated cells. These data demonstrated that one of mechanisms of AGEs-induced eNOS down-regulation is the decrease of eNOS mRNA stability in AGEs-treated human endothelial cells.

Cardiovascular disease is a multifactorial disease. One of the major reasons is endothelial dysfunction that is characterized by a decrease in NO bioactivity, with a concomitant increase in superoxide formation, despite the observation that eNOS mRNA and protein levels are maintained or even increased. For example, endothelial function was impaired whereas eNOS protein expression was increased in response to hyperglycemia [18, 19] or advanced age [17, 20]. eNOS overexpression in apolipoprotein E knockout mice was reported to accelerate the development of atherosclerosis [21]. These findings indicate that alteration of eNOS itself can be a significant cause for endothelial dysfunction, and sufficient expression of eNOS protein alone does not guarantee bioavailability of NO. Madamanchi et al. demonstrated that ROS was the key mediators for vascular inflammation and atherogenesis [22]. Human investigations support the oxidative stress hypothesis of atherogenesis [22, 23]. This is further supported by impaired vascular function and enhanced atherogenesis in animal models that have deficiencies in internal antioxidant enzymes [24]. In the present study, we found that AGEs induces a significant increase of O_2_^−^ in HCAECs, which could be one of the mechanisms for AGEs-induced eNOS dysfunction.

To explore where increased O_2_^−^ originated from in AGEs-treated cells, we verified that the decrease of mitochondrial membrane potential could cause the increase of O_2_^−^ in AGEs-treated cells. In the meantime, this result suggests that AGEs may induce mitochondrial dysfunction, which in turn, may be partially responsible for the increase in O_2_^−^ production detected in AGEs-treated cells. Interestingly, treatment with TTFA, a mitochondrial inhibitor, effectively blocked AGEs-induced the increase of O_2_ production and the decrease of eNOS. Clearly, treatment with AGEs substantially increases NOX activity, whereas it decreases CAT and SOD activities. This may impair the internal cellular response to oxidative stress in AGEs treated cells. Thus AGEs-increased O_2_^−^ production may result from mitochondrial dysfunction and compromised cellular redox enzymes. Similar results were obtained from the antioxidants SeMet and Specific O_2_^−^ scavenger MnTBAP, which blocked AGEs’s effects on O_2_^−^ production in AGEs-treated cells.

Beside above reason on O_2_^−^ production, xanthine oxidase and lipoxygenases. SOD facilitate the decrease of O_2_^−^ by converting O_2_^−^ to of H_2_O_2_ [25], and then CAT [26] and glutathione peroxidase (GPX) [27] coordinate the conversion of H_2_O_2_ to water. SeMet is the form reported to be the major component of dietary selenium, and undergoes an intramolecular transsulfuration reaction to form selenocysteine [28]. It is able to directly interact with some oxidant molecules or oxidant-generating ions, which, in turn, increases the activity of internal antioxidant enzymes GPX and thioredoxin reductase [29, 30]. These two antioxidants effectively blocked AGEs-induced eNOS downregulation and ROS production. Thus, our findings provide a suggestion that antioxidant therapy may be an effective strategy in treating vascular diseases related to AGEs.

Inflammatory also play an important role in the development of vascular damage associated to hyperglycemia [31, 32]. During inflammation, inducible nitric oxide synthase activation generates an overabundance of NO in the circulation and induced the cardiovascular dysfunction. High levels of NO availability without inflammatory stress can promote insulin resistance [33]. Nair M et al. shown that endoplasmic reticulum stress induced by glyLDL is possibly involved in eNOS downregulation [34]. In addition, insulin plays an important role in the regulation of vascular homeostasis and maintenance of endothelial function. Insulin signaling might cause activation of two separate and parallel pathways: PI3K/AKT/eNOS and Ras/Raf/MAPK pathways [35]. AKT phosphorylates eNOS at Ser1177, resulting in increased nitric oxide production and vasodilation. The MAPK pathway results in endothelin-1 production and vasoconstriction and mitogenic effects [19].

MAPKs are important in regulating cell growth, migration, and differentiation in response to various extracellular stimuli [11]. The pattern of MAPK activation in response to oxidative stress varies depending on the oxidant strength and cell type. There is increasing evidence that the engagement of AGEs by RAGE mainly recruits MAPKs including p44/42 MAPK, p38 MAPK, and Jun N-terminal kinase (JNK) and a downstream activation of the transcription factor NF-kappaB for induction of proinflammatory and procoagulatory gene expression [36, 37]. In the study, we demonstrated that p38 and ERKl/2 were activated in response to AGEs stimulation, and that the p38 inhibitor SB-239063 and the ERKl/2 inhibitor PD-98059 effectively blocked AGEs-induced eNOS downregulation and ROS production in HCAECs.

Although different clinical studies reported different serum or plasma levels of AGEs due to different measurement procedures and conditions, human AGEs levels usually increase in patients with diabetes. Our data demonstrated that compared with healthy individuals, AGEs levels significantly increased in diabetic patients. In the current study, we have used a much higher concentration (100 or 200 μg/l) for in vitro experiments than those in human serum or plasma levels. The major reason for this choice is considering that similar concentrations of AGEs are used in the previous discoveries of AGEs functions and underlying mechanisms from in vitro experiments in endothelial cells [38, 39].

Thus, the data gene rated from the current study is comparable with those in the previous publications. In addition, local concentrations of AGEs at the vascular lesion site may be much higher than those in patients’ serum or plasma.

## Conclusions

Our study demonstrates that AGEs, at concentrations found in diabetes significantly decrease eNOS expression and NO production through oxidative stress, p38 and ERK l/2 MAPK activation in HCAECs. AGEs induced mitochondrial dysfunction and unbalanced cellular redox enzymes may be the underlying mechanisms of increased ROS production. These data, combined with the finding that AGEs expression is increased in human atherosclerotic tissues, support the hypothesis that AGEs may contribute to vascular disease through endothelial dysfunction. Antioxidants SeMet and MnTBAP potentially hold clinical applications in blocking AGEs-induced endothelial dysfunction, thereby preventing vascular disease.

## Additional file

**Additional file 1: Figure S1.** Effects of AGEs on eNOS protein levels and NOS activity in HCAECs. Western blot analysis. Cells were treated with 100 μg/l AGEs for 24 h. Representative bands of p-eNOS (SER1177) and β-actin staining and quantitation of band density ratios. Full-length blots are presented. **P* < 0.05 compare with control, *n* = 3 experiments. Data are means and SE of multiple experiments (n).

## Abbreviations

PI3K: phosphatidylinositol-4,5-bisphosphate 3-kinase
AKT: protein kinase B
eNOS: endothelial nitric oxide synthase
NO: nitric oxide
MAPK: mitogenactivated protein kinase
ERK1/2: extracellular signal-regulated kinase 1/2
JNK: jun-amino(N)-terminal kinase
HCAECs: human coronary artery endothelial cells
