# Supplementary material for: Advanced glycation end-products decreases expression of endothelial nitric oxide synthase through oxidative stress in human coronary artery endothelial cells
Source: Cardiovasc Diabetol. 2017 Apr 20;16:52. doi: 10.1186/s12933-017-0531-9 (PMC5397770; doi:10.1186/s12933-017-0531-9)
Supplement: Supplementary file 1 — Additional file 1: Figure S1. Effects of AGEs on eNOS protein levels and NOS activity in HCAECs. Western blot analysis. Cells were treated with 100 μg/l AGEs for 24 h. Representative bands of p-eNOS (SER1177) and β-actin staining and quantitation of band density ratios. Full-length blots are presented. *P < 0.05 compare with control, n = 3 experiments. Data are means and SE of multiple experiments (n). [file 12933_2017_531_MOESM1_ESM.pdf]

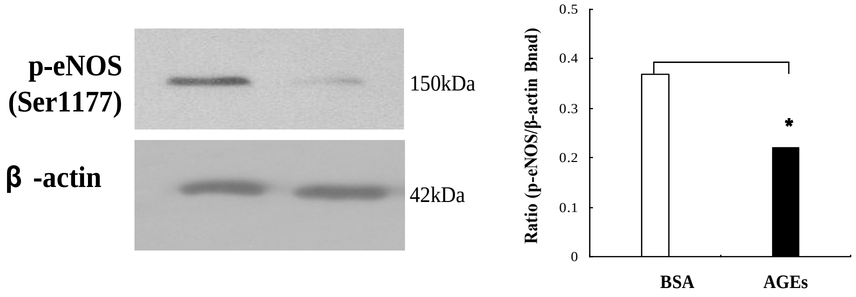

Supplementary Fig. S1 Effects of AGEs on eNOS protein levels and NOS activity in HCAECs. Western blot analysis. Cells were treated with 100ug/l AGEs for 24 h. Representative bands of p-eNOS (SER1177) and β-actin staining and quantitation of band density ratios. Full-length blots are presented. **P* < *0.05* compare with control, *n* = *3* experiments. Data are means and SE of multiple experiments (n).
